# SkateBase, an elasmobranch genome project and collection of molecular resources for chondrichthyan fishes

**DOI:** 10.12688/f1000research.4996.1

**Published:** 2014-08-12

**Authors:** Jennifer Wyffels, Benjamin L. King, James Vincent, Chuming Chen, Cathy H. Wu, Shawn W. Polson

**Affiliations:** 1 Department of Computer and Information Sciences, Center for Bioinformatics and Computational Biology, University of Delaware, Newark, DE, 19711, USA; 2 Mount Desert Island Biological Laboratory, Salisbury Cove, ME, 04672, USA; 3 Vermont Genetics Network, University of Vermont, Burlington, VT, 05405, USA

## Abstract

Chondrichthyan fishes are a diverse class of gnathostomes that provide a valuable perspective on fundamental characteristics shared by all jawed and limbed vertebrates. Studies of phylogeny, species diversity, population structure, conservation, and physiology are accelerated by genomic, transcriptomic and protein sequence data. These data are widely available for many sarcopterygii (coelacanth, lungfish and tetrapods) and actinoptergii (ray-finned fish including teleosts) taxa, but limited for chondrichthyan fishes.  In this study, we summarize available data for chondrichthyes and describe resources for one of the largest projects to characterize one of these fish, *Leucoraja erinacea*, the little skate.  SkateBase ( http://skatebase.org) serves as the skate genome project portal linking data, research tools, and teaching resources.

## Introduction

Chondrichthyan fishes are composed of two subclasses, Holocephali and Elasmobranchii. Holocephalans are the more basal of the pair having first appeared more than 400 million years ago and include a single surviving order, Chimaeriformes, the chimaeras, with 39 extant species ^1^. Elasmobranchs appeared approximately 350 million years ago and include more than 1000 species of sharks, skates, and rays ^2^. Chondrichthyan fishes occupy a pivotal position at the base of the vertebrate phylogenetic tree. For research that includes an evolutionary component, representation of this diverse class affords a valuable perspective to evaluate all vertebrates.

Chondrichthyan fishes are circumglobal in distribution and occupy a wide range of ecological habitats. Their life history parameters are equally disparate but in general chondrichthyans are slow growing and late maturing fishes with an increased risk of extinction ^3– 5^. Fecundity is as few as 1 or 2 for viviparous species such as the sand tiger shark, *Carcharias taurus* ^6^ and as high as 300 for the whale shark, *Rhincodon typus* ^7^. They are of economic importance for fisheries as well as ecotourism. Management and assessment of stock is essential to ensure both ecotourism interests and food resources remain sustainable ^8^. Management of fish populations has increasingly relied on molecular tools to investigate population structure, properly identify species, and compliance with fishing quotas ^9– 13^.

Elasmobranchs have been used as a model for biomedical research for more than 100 years. Elasmobranchs, like other cartilaginous fishes, exhibit many fundamental vertebrate characteristics, including a neural crest, jaws and teeth, an adaptive immune system, and a pressurized circulatory system. The skate is a powerful comparative model to study biological processes shared among jawed and limbed vertebrates such as development ^14– 16^, renal physiology ^17– 20^, immunology ^21– 26^, toxicology ^27^, neurobiology ^28^, and wound healing and regeneration ^29^. They are the most ancient vertebrates to posses an adaptive immune system that generates antibodies using a V(D)J combinatorial mechanism ^30^. Phylogenetically, cartilaginous fishes are the first vertebrates to possess a thymus, a central lymphoid organ that provides a microenvironment for the development of T cells ^31^. The thymus shares a common organization with more derived vertebrates containing cortical and medullary regions ^32, 33^.

In addition to shared physiological characteristics, the diversity of specializations between species allows investigations of evolution within a single clade. For example, elasmobranchs use a plethora of reproductive strategies that span the full range of maternal investment from placental viviparity to strict lecitrophic oviparity. Besides sexual reproduction, captive elasmobranchs are capable of asexual parthenogenesis ^34– 36^. Of these reproductive mechanisms, the most tractable for research purposes is oviparity. Approximately 43% of chondrichthyans utilize oviparity including all Chimaeriformes, Heterodontiformes (bullhead sharks), Rajoidae (skates) and Scyliorhinidae (catsharks) ^37^. Many species can be maintained in captivity and will breed and lay eggs throughout an annual season ^38^. Artificial insemination has been reported for two oviparous species, the clearnose skate, *Raja eglanteria* ^39^, and the cloudy catshark, *Scyliorhinus torazame* ^40^. Additionally, sperm storage allows wild caught females to lay eggs for several years without requiring males or captive mating events ^41^.

*Leucoraja erinacea*, the little skate, was chosen for a genome sequencing project to represent this clade of fishes because of their use as a biomedical model, experimental tractability, genome size, existing sequence data, and northeast regional distribution. The sequencing project is an ongoing effort of the North East Bioinformatics Collaborative (NEBC) of the North East Cyberinfrastructure Consortium (NECC), composed of the bioinformatics core facilities from Delaware, Maine, New Hampshire, Rhode Island, and Vermont funded by National Institutes of Health (NIH) Institutional Development Awards (IDeA) and/or National Science Foundation (NSF) Experimental Program to Stimulate Competitive Research (EPSCoR) programs.

## Existing resources

**Figure 1.**
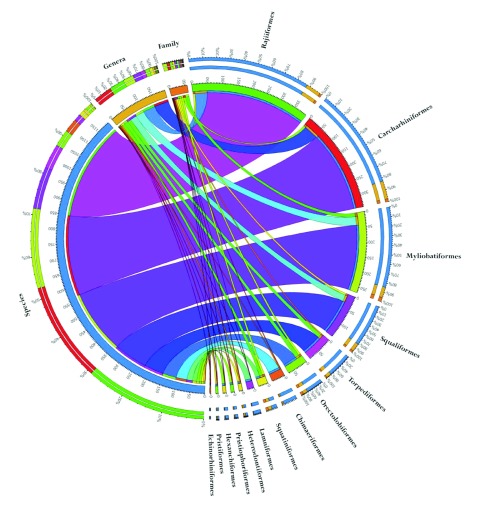
Species distribution within chondrichthyan orders. There is a single order of Holocephalans, Chimaeriformes, and 13 orders of elasmobranchs. The distribution of chondrichthyan species in each of the 14 orders is shown relative to the total number of species, genera and families for the clade. The batoids are composed of 4 orders, Rajiiformes, Myliobatiformes, Torpidiformes, and Rhinopristiformes, and contain 54% of extant chondrichthyan species. Sharks are broadly divided into two super orders, Galeomorphii and Squalomorphii that together include the remaining 9 orders and 43% of extant chondrichthyan species.

There is a single order of holocephalans and 13 orders of elasmobranchs. The distribution of species in orders, families and genera is shown in Figure 1. The batoids are composed of 4 orders, Rajiiformes, Myliobatiformes, Torpidiformes, and Rhinopristiformes, and contain 54% of extant chondrichthyan species. Sharks are broadly divided into two super orders, Galeomorphii and Squalomorphii that together account for 43% of extant chondrichthyan species. The galean sharks include 4 orders: Heterdontiformes, Orectolobiformes, Lamniormes and Carcharhiniformes, and represent 30% of extant chondrichthyan species. Squalean sharks are composed of 4 orders: Squaliformes, Squatiniformes, Pristophoriformes, and Hexanchiformes, comprising 13% of extant chondrichthyan species. Among individual orders, Rajiiformes, the skates, have the most species (345) followed by Carcharhiniformes, the ground sharks (283) and Myliobatiformes (226) ^2^. These ‘big three’ orders contain 854 species, 72% of extant chondrichthyans.

**Figure 2.**
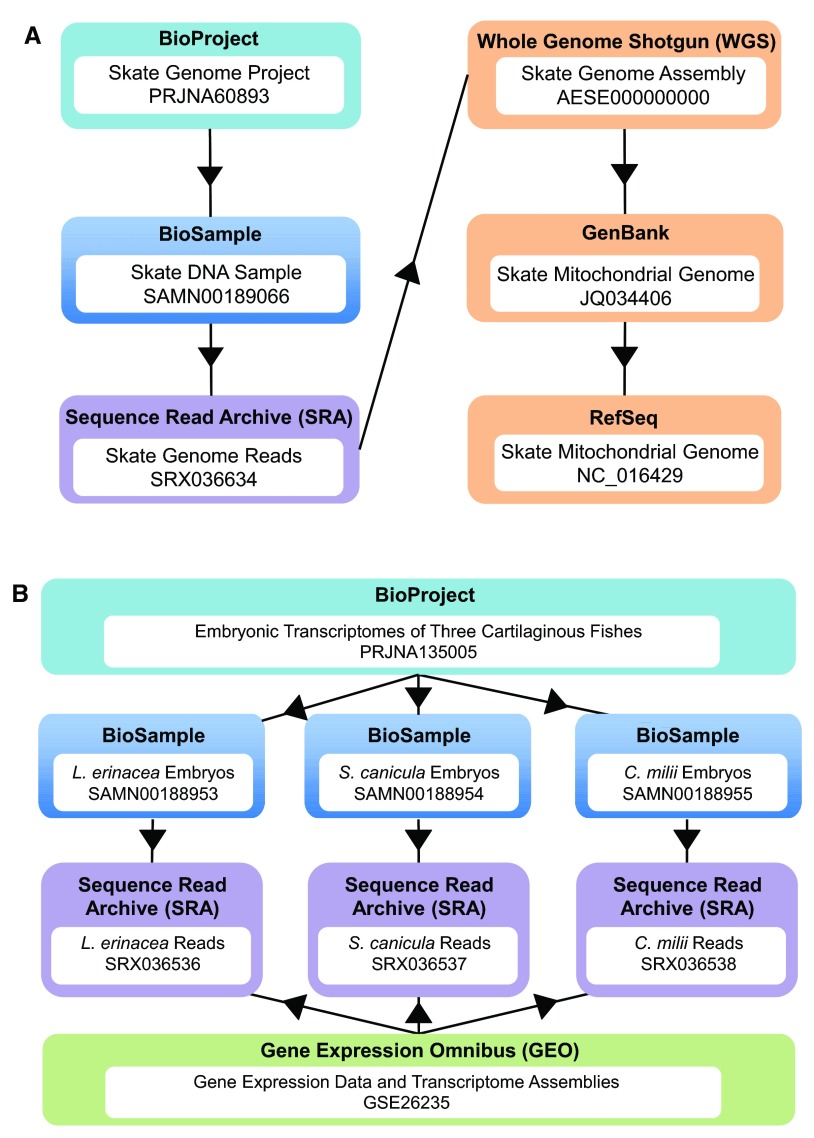
Representation of SkateBase data within the The National Center for Biotechnology Information (NCBI) databases. **A**. The little skate genome project is represented as a BioProject entry that connects all samples and data thematically. A BioSample record describes the DNA sample that was used for genome sequencing that was generated from a single stage 32 skate embryo. The SRA catalogs the unassembled Illumina genome sequence data. The Whole Genome Shotgun (WGS) database contains the contiguous sequences from shotgun sequencing projects. The assembled and annotated mitochondrial genome was deposited in GenBank and subsequently included in the NCBI Reference Sequence Database (RefSeq). **B**. The project to characterize the embryonic transcriptomes of *L. erinacea*, *C. milii* and *S. canicula is* represented in a BioProject entry. Three BioSample entries, one for each species, lead to three SRA datasets. The transcriptome data is represented also in the Gene Expression Omnibus (GEO), a database of high-throughput functional genomic data derived from microarrays and next-generation sequencing technologies.

Chondrichthyan conservation, management, and research all benefit from easily accessible and well-documented molecular resources. The organization of data and metadata in archival databases is critically important for efficient use of large and complex datasets. The International Nucleotide Sequence Database Collaboration (INSDC) is composed of three large public nucleotide repositories, DNA Data Bank of Japan (DDBJ), European Molecular Biology Laboratory-European Bioinformatics Institute (EMBL-EBI), and GenBank at the National Center for Biotechnology Information (NCBI). Recently, two new NCBI database projects were initiated to collect details of samples, BioSample, and project data, BioProject, and propagate the metadata to all associated database entries in an effort to expand the use of already existing and rapidly expanding molecular resources ^42^. Figure 2 illustrates the relationship between BioProject, BioSample and the sequence data for SkateBase. Because the BioProject and BioSample databases were established in 2012, not all existing datasets have metadata or details of the biological source to populate a BioSample and BioProject entry. When available, BioProject and BioSample hyperlinks are included for Sequence Read Archive (SRA), Expressed Sequence Tag (EST) and Genome Survey Sequence (GSS) datasets in the tables below.

**Figure 3.**
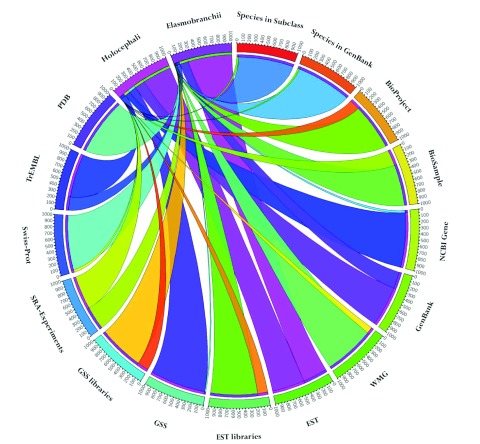
Holocephalan and elasmobranch resources in public nucleotide and protein databases. The distribution of data for Holocephalii (chimaeras) and elasmobranchii (sharks and rays) subclasses of chondrichthyan fishes does not always reflect their species distribution. The number of species represented in GenBank is representative of the actual species distribution but the amount of data in GenBank is not. Holocephalan data forms the majority of the NCBI Gene, GenBank, Genome Survey Sequence (GSS) and UniProt TrEMBL databases. The number of Short Reach Archive (SRA) experiments and EST sequences in nearly equal for each subclass and the remaining databases are primarily populated by elasmobranch data.

Table 1 is a summary of chondrichthyan sequence data in NCBI databases, UniProtKB, and the Protein Data Bank (PDB) with *L. erinacea*, *Callorhinchus milii* and *Scyliorhinus canicula*, the three species featured at SkateBase listed individually. The distribution of holocephalans and elasmobranchs in public databases is illustrated in Figure 3. Despite the majority of species belonging to Elasmobranchii, the GenBank, UniProtKB/TrEMBL, and Gene databases are dominated by chimaera data derived from the genome sequence of the elephant shark, *C. milii* ^43^. Elasmobranch data predominates in UniProtKB/Swiss-Prot, PDB, BioProject and BioSample databases as well as the number of whole mitochondrial genomes (WMG) in GenBank. The EST and SRA databases are nearly equally split between the two subclasses.

**Table 1.** Chondrichthyan molecular sequence data in public databases.

	National Center for Biotechnology Information (NCBI) databases ^1^														
					GenBank									UniProtKB ^2^		
	Taxonomy	BioProject	BioSample	Gene	GenBank	WMG	EST	EST lib	GSS	GSS lib	WGS (Mbp)	GEO ^3^	SRA	Swiss- Prot	TrEMBL	PDB
Chondrichthyes	7777	16	75	21069	55810	72	192948	33	28497	5	2492.3	3	22	276	26485 ^*^	178
Holocephali	7863	3	21	20201	39512	8	109965	6	27944	1	936.9	1	13	12	20170	0
*C. milii*	7868	3	21	20110	39232	1	109965	6	27944	1	936.9	1	13	3	19989	0
Elasmobranchii	7778	13	54	868	16273	64	82983	27	553	4	1555.4	2	9	264	6299	178
*L. erinacea*	7782	3	7	13	284	1	31167	5	0	0	1555.4	1	2	6	123	0
*S. canicula*	7830	2	8	13	645	1	1600	7	0	0	0	1	1	38	283	1

(WMG) whole mitochondrial genome, (EST) Expressed Sequence Tags, (lib) libraries (GSS) Genome Survey Sequences, (GEO) Gene Expression Omnibus, (WGS) Whole Genome Shotgun, (SRA) Sequence Read Archive, (WMG) whole mitochondrial genomes, (PDB) Protein Data Bank, * includes 16 unidentified fin entries

^1^ NCBI databases accessed July 25, 2014, ^2^ Release 2014_07 of 09-Jul-2014, ^3^ GEO sample accessions

## Chondrichthyan genomes

Currently there are multiple efforts to sequence an elasmobranch genome in various stages of completion ( Table 2); however, only the skate genome project currently has data publically available. Efforts to sequence the whale shark are underway at the Georgia Aquarium and Emory University (personal communication, Alistair Dove, Georgia Aquarium). Genoscope leads a project to sequence the genome of another oviparous elasmobranch, the catshark, *S. canicula*. The current assembly is described in Table 2. A second version of the catshark genome with 200x coverage, including mate pair sequencing, is in progress (personal communication, Sylvie Mazan, French National Centre for Scientific Research). Among holocephalans, the genome of the elephant shark, *C. milii*, was first described in a 1.4x coverage assembly in 2006 ^44^. With continued sequencing the assembly coverage is currently 19.25x and data has been made available through the project website ( http://esharkgenome.imcb.a-star.edu.sg/) and Genbank ^43^.

**Table 2.** Chondrichthyan genome sequencing projects.

	Website	Genome size (Gb)	Coverage	Contigs	N50 (bp)	Platform	Facility	Genbank	Data ^1^	BioProject	BioSample	Date
Holocephali												
*Callorhinchus milii*	esharkgenome	0.910	19.25x	21,203	1466	Sanger & 454	IMCB	AAVX02000000	244 M	PRJNA236996	SAMN00000800	20-Dec-13 ^*^
Elasmobranchii												
*Leucoraja erinacea*	skatebase.org	3.42	26x	2,62,365	665	Illumina PE	NECC	AESE010000000	105 G	PRJNA60893	SAMN00189066	22-Dec-11
*Scyliorhinus canicula*	-	3.5	32x	3,449,662	1,292	Illumina PE	Genoscope-CEA	-	-	-	-	-
*Rhincodon typus*	-	3.44(est.)	35x			Illumina & 454	Emory University & Georgia Aquarium	-	-	PRJNA255419	SAMN02918461 SAMN02918462	16-Jul-14

^1^ (M) Mega or (G) Giga base pairs; (PE) paired end; (est) estimated; (ICMB) Institute of Molecular and Cell Biology, A*STAR, (NECC) North East Cyberinfrastructure Consortium

* replaced original sequence data GenBank AAVX00000000.1 (1.4x coverage) released 20-DEC-2006

A powerful resource for characterizing genomes is large-insert clone libraries where each clone contains a large (~100kb) genomic region. Bacterial artificial chromosome (BAC) and P1-derived artificial chromosome (PAC) libraries are DNA constructs within a plasmid used to transform bacteria. As the bacteria grow the inserted DNA is amplified and subsequently isolated and sequenced. BACs are beneficial for genome sequencing projects because the insert size can be very large, nearly 350 kb, facilitating assembly post-sequencing. BAC/PAC libraries were built for several chondrichthyan species including the nurse shark, *Ginglymostoma cirratum* ^45^; elephant shark, *C. milli* ^46^; little skate, *L. erinacea* ^47^; horn shark, *Heterdontus francisci* ^48^; dogfish shark, *Squalus acanthias* ^49, 50^; and catshark, *S. canicula* ^51^. These libraries were used to successfully characterize a handful of genomic regions such as little skate *HoxA* cluster ^47, 52^, horn shark *Hox A* and *D* clusters ^53^, catshark *HoxA*, *B* and *D* clusters ^51, 52^, *C. milii* HoxA-D clusters ^54^, immunoglobulin receptor IgW C regions ^30^ and neurohypophysial gene loci ^46^.

## RNA databases

Transcriptome sequencing seeks to characterize all genes expressed in a tissue or set of tissues in a sample. Technologies to identify the complete RNA transcript sequence have developed from studies of a small number of transcripts to comprehensive characterizations. The application of large-scale cDNA cloning of Expressed Sequence Tags (ESTs) gave initial characterizations of 5-prime and/or 3-prime ends of transcripts in several elasmobranchs including *L. erinacea* and *S. acanthias* ( Table 3). EST sequence data are available in the EST divisions of the GenBank, EMBL and DDBJ databases that make up the INSDC. cDNA clones and their sequences from these EST projects have enabled the complete characterization of the full-length cDNA sequence of several genes. In the last five years, high-throughput RNA sequencing (RNA-Seq) has been applied to comprehensively examine the complete sequence of transcripts in tissues of cartilaginous fishes. Among the most valuable RNA-Seq datasets are those from whole late-stage embryos following organogenesis. Our project has generated these datasets for *L. erinacea*, *S. canicula* and *C. milii* ^52^. Public RNA-Seq data sets can be found in the NCBI Gene Expression Omnibus and Short Read Archive (SRA) databases or the EBI ArrayExpress and European Nucleic Acid (ENA) archives ( Table 3 and Table 4).

**Table 3.** National Center for Biotechnology Information (NCBI) Expressed Sequence Tags (EST) and Genome Survey Sequences (GSS) databases (release 130101): Chondrichthyan sequence data.

BioSample	BioSample Description	Library ID	Organism	Sample age/sex	Sample type	ESTs	Facility ^1^	Date
Holocephali								
Chimaeriformes								
182978	Whole-genome shotgun library of the elephant shark (aka elephant fish)	GSS: LIBGSS_009694	*Callorhinchus milii*	-	testis	27944	IMCB	2004
1000678	Elephant shark full- length cDNA library from testis	EST: LIBEST_027873	*Callorhinchus milii*	-	testis	29234	IMCB	2012
1000677	Elephant shark full- length cDNA library from spleen	EST: LIBEST_027872	*Callorhinchus milii*	-	spleen	16664	IMCB	2012
1000676	Elephant shark full- length cDNA library from liver	EST: LIBEST_027871	*Callorhinchus milii*	-	liver	16573	IMCB	2012
1000675	Elephant shark full- length cDNA library from kidney	EST: LIBEST_027870	*Callorhinchus milii*	-	kidney	19246	IMCB	2012
1000674	Elephant shark full- length cDNA library from intestine	EST: LIBEST_027869	*Callorhinchus milii*	-	intestine	12146	IMCB	2012
1000673	Elephant shark full- length cDNA library from gills	EST: LIBEST_027868	*Callorhinchus milii*	-	gills	16012	IMCB	2012
Elasmobranchii: Batoids (rays and skates)								
Torpediformes								
158311	Torpedo marmorata electric organ	EST: LIBEST_003755	*Torpedo marmorata*	-	electric organ	8	CNRS	2000
158310	Torpedo marmorata electric lobe	EST: LIBEST_003754	*Torpedo marmorata*	-	electric lobe	26	CNRS	2000
157461	pFL61-TEL	EST: LIBEST_002905	*Torpedo marmorata*	-	electric lobe	2	CNRS	2000
157406	pFL61-EL	EST: LIBEST_002849	*Torpedo marmorata*	-	electric lobe	5	CNRS	2000
154382	Torpedo californica electric organ	EST: LIBEST_020696	*Torpedo californica*	-	electric organ	10185	Children’s National Medical Center, USA	2006
Rajiformes								
175126	Little Skate Multiple Tissues, Normalized	EST: LIBEST_015890	*Leucoraja erinacea*	adult	mixed ^a^	5698	MDIBL	2004
176484	Little Skate Liver, Normalized	EST: LIBEST_017626	*Leucoraja erinacea*	adult	liver	6016	MDIBL	2005
165533	Little Skate embryo cell line 1 (LEE-1): 5' sequences	EST: LIBEST_022984	*Leucoraja erinacea*	embryonic cell line	stage 28	4825	MDIBL	2006
154366	Little skate embryo tissues; 5' sequences	EST: LIBEST_020422	*Leucoraja erinacea*	embryo	stage 19, 20, 25	5600	MDIBL	2006
166469	Skate Multiple Tissues, Normalized	EST: LIBEST_023576	*Leucoraja erinacea*	adult	mixed ^a^	9028	MDIBL	2008
Elasmonbranchii: Selachii (sharks)								
Carcharhiniformes								
168576	Dogfish testis - round spermatids zone (SSH)	EST: LIBEST_025578	*Scyliorhinus* *canicula*	adult	testis	20	Caen University	2009
168575	Dogfish testis - spermatogonia zone (SSH)	EST: LIBEST_025577	*Scyliorhinus* *canicula*	adult	testis	12	Caen University	2010
222714	Scyliorhinus canicula juvenile library	EST: LIBEST_026904	*Scyliorhinus* *canicula*	juvenile	5 days post-hatch	56	enoscope-CEA	2011
222713	Scyliorhinus canicula embryonic, stages 9–15 library	EST: LIBEST_026903	*Scyliorhinus* *canicula*	embryo	stages 9–15	628	Genoscope-CEA	2011
222712	Scyliorhinus canicula embryonic, stages 19–25 library	EST: LIBEST_026902	*Scyliorhinus* *canicula*	embryo	stages 19–25	772	Genoscope-CEA	2011
222711	Scyliorhinus canicula embryonic, stages 19–24 library	EST: LIBEST_026901	*Scyliorhinus* *canicula*	embryo	stages 19–24	33	Genoscope-CEA	2011
222710	Scyliorhinus canicula adult brain library	EST: LIBEST_026900	*Scyliorhinus* *canicula*	adult	brain	79	Genoscope-CEA	2011
699400	cloudy catshark embryo cDNA library	EST: LIBEST_027410	*Scyliorhinus* *torazame*	embryo	stage 31	2942	RIKEN	2011
Orectolobiformes								
183175	GC__Ba	GSS: LIBGSS_009945	*Ginglymostoma* *cirratum*	adult	red blood cells	178	University of Arizona	2005
184343	shark whole genome shotgun library 2	GSS: LIBGSS_011249	*Chiloscyllium* *plagiosum*	female	ventral fin	177	Tgen	2008
184342	shark whole genome shotgun library 1	GSS: LIBGSS_011248	*Chiloscyllium* *plagiosum*	female	ventral fin	194	Tgen	2008
166749	Shark liver regeneration	EST: LIBEST_023789	*Chiloscyllium* *plagiosum*	adult	liver	2103	BGI	2008
176026	cDNA library of Shark hepatic regeneration tissues	EST: LIBEST_017019	*Chiloscyllium* *plagiosum*	none	Hour 24 after 2/3 partial hepatectomy	17	CPU	2005
254067	Toll like receptor ligand induced Spleen	EST: LIBEST_027180	*Chiloscyllium* *griseum*	male	spleen	1051	MVC	2011
254066	Spleen of Chiloscyllium griseum	EST: LIBEST_027179	*Chiloscyllium* *griseum*	male	spleen	1000	MVC	2011
1797282	Suppressive subtractive hybridization library from peptidoglycan induced spleen of the shark	EST: LIBEST_028031	*Chiloscyllium* *griseum*	male	spleen	315	MVC	2012
Squaliformes								
175664	Dogfish Shark Multiple Tissues, Normalized	EST: LIBEST_016552	*Squalus acanthias*	adult	mixed ^b^	15078	MDIBL	2004
176998	Dogfish Shark Embryo-derived Cell Line SAE, Normalized	EST: LIBEST_018195	*Squalus acanthias*	embryonic cell line	embryo with external yolk sac	5824	MDIBL	2005
154362	Spiny dogfish shark rectal gland EST library	EST: LIBEST_020417	*Squalus acanthias*	-	rectal gland	5085	MDIBL	2006
150616	Dogfish Shark Rectal Gland, Normalized	EST: LIBEST_020023	*Squalus acanthias*	adult	rectal gland	6575	MDIBL	2006
Hexanchiformes								
178140	Hexanchus griseus DNA (Hunter C)	GSS: LIBGSS_003277	*Hexanchus* *griseus*	-	-	4	HGMP-RC	2001

^1^ (ICMB) Institute of Molecular and Cell Biology, A*STAR, (HGMP-RC) Human Genome Mapping Project Resource Centre, Hinxton, (Tgen) Translational Genomics Research Institute AZ, USA, (CNRS) National Center for Scientific Research, France, (MDIBL) Mount Desert Island Biological Laboratory, (CPU) China Pharmaceutical University, (MVC) Madras Veterinary College, TANUVAS, (BGI) Beijing Genomics Institute (SSH) Suppressive subtractive hybridization; (mixed ^a^) liver, kidney, brain, testis, ovary, gill, heart, spleen, rectal gland; (mixed ^b^) rectal gland, kidney, brain, testis, ovary, gill, intestine, heart, spleen

**Table 4.** National Center for Biotechnology Information (NCBI) Sequence Read Archive (SRA) database: Chondrichthyan sequence data.

BioProject	BioSample	SRA description	SRA	Organism	Age	Sample type	Platform ^1^	Data ^2^	Facility ^3^	Date
Holocephali										
PRJNA18361	SAMN00000800	454 sequencing of Callorhinchus milii genomic fragment library	SRX001870 ^*^	*Callorhinchus milii*	adult	testis	LS454	244.9 M	IMCB	2008
PRJNA135005	SAMN00188955	GSM643959: Callorhinchus milii pooled Stage 32 embryos	SRX036538	*Callorhinchus milii*	embryos	stage 32	Illumina SE	3.3 G	MDIBL	2011
PRJNA168475	SAMN02699939	Illumina sequencing of elephant shark thymus RNA	SRX220387	*Callorhinchus milii*	-	thymus	Illumina PE	9.7 G	IMCB	2013
PRJNA168475	SAMN02699938	Illumina sequencing of elephant shark testis RNA	SRX154861	*Callorhinchus milii*	-	testis	Illumina PE	7.3 G	IMCB	2013
PRJNA168475	SAMN02699937	Illumina sequencing of elephant shark spleen RNA	SRX154860	*Callorhinchus milii*	-	spleen	Illumina PE	6.3 G	IMCB	2013
PRJNA168475	SAMN02699936	Illumina sequencing of elephant shark ovary RNA	SRX154859	*Callorhinchus milii*	-	ovary	Illumina PE	7.9 G	IMCB	2013
PRJNA168475	SAMN02699935	Illumina sequencing of elephant shark liver RNA	SRX154858	*Callorhinchus milii*	-	liver	Illumina PE	16.7 G	IMCB	2013
PRJNA168475	SAMN02699934	Illumina sequencing of elephant shark muscle RNA	SRX154857	*Callorhinchus milii*	-	muscle	Illumina PE	11.1 G	IMCB	2013
PRJNA168475	SAMN02699933	Illumina sequencing of elephant shark kidney RNA	SRX154856	*Callorhinchus milii*	-	kidney	Illumina PE	9 G	IMCB	2013
PRJNA168475	SAMN02699932	Illumina sequencing of elephant shark intestine RNA	SRX154855	*Callorhinchus milii*	-	intestine	Illumina PE	11.2 G	IMCB	2013
PRJNA168475	SAMN02699931	Illumina sequencing of elephant shark heart RNA	SRX154854	*Callorhinchus milii*	-	heart	Illumina PE	6.9 G	IMCB	2013
PRJNA168475	SAMN02699930	Illumina sequencing of elephant shark gills RNA	SRX154852	*Callorhinchus milii*	-	gills	Illumina PE	5.4 G	IMCB	2013
PRJNA168475	SAMN02699929	Illumina sequencing of elephant shark brain RNA	SRX154851	*Callorhinchus milii*	-	brain	Illumina PE	10.5 G	IMCB	2013
Elasmobranchii										
PRJNA60893	SAMN00189066	Initial Characterization of Leucoraja erinacea Genome Using 500bp Paired- End Sequencing	SRX036634 ^*^	*Leucoraja erinacea*	embryo	stage 32	Illumina PE	105 G	NECC	2011
PRJNA135005	SAMN00188953	GSM643957: Leucoraja erinacea pooled Stage 20–29 embryos	SRX036536	*Leucoraja erinacea*	embryos	stage 20–29	Illumina SE	3.8 G	MDIBL	2011
PRJNA135005	SAMN00188954	GSM643958: Scyliorhinus canicula pooled Stage 24–30 embryos	SRX036537	*Scyliorhinus canicula*	embryos	stage 24–30	Illumina SE	3.9 G	MDIBL	2011	
PRJDA61447	SAMD00003843	Torazame EST	DRX000491	*Scyliorhinus torazame*	embryos	stage 23–31	LS454	43.6 M	RIKEN	2011
PRJNA177971	SAMN01915239	Carcharodon carcharias cDNA Illumina sequence reads	SRX228421	*Carcharodon carcharias*	juvenile	heart	Illumina SE	7.9 G	Cornell	2013
PRJNA177971	SAMN01915239	Carcharodon carcharias heart transcriptome	SRX228332	*Carcharodon carcharias*	juvenile	heart	LS454	408.4 M	Cornell	2013
PRJNA183979	SAMN01831510	Illumina sequencing of Nurse Shark thymus transcripts	SRX219866	*Ginglymostoma cirratum*	-	thymus	Illumina PE	12 G	IMCB	2013
PRJNA183979	SAMN01831509	Illumina sequencing of Nurse Shark spleen transcripts	SRX219865	*Ginglymostoma cirratum*	-	spleen	Illumina PE	11.2 G	IMCB	2013
PRJNA240112	SAMN02673223	Neotrygon kuhlii barb venom gland transcriptome	SRX481088	*Neotrygon kuhlii*	-	barb venom gland	Illumina PE	84.3 M	LSTM	2014

* genomic data; ^1^ (SE) single end or (PE) paired end; ^2^ (M) Mega or (G) Giga base pairs

^3^ (MDIBL) Mount Desert Island Biological Laboratory, (ICMB) Institute of Molecular and Cell Biology, A*STAR, (LSTM) Liverpool School of Tropical Medicine, (NECC) North East Cyberinfrastructure Consortium

## Mitochondrial genomes

Individual mitochondrial genes such as cytochrome c oxidase subunit I (CO1 or COX1) and NADH-ubiquinone oxidoreductase chain 2 (NADH2 or MT-ND2) have been used extensively to construct molecular phylogenies ^55– 57^. The Fish barcode of life (FISH-BOL) a working group of the International Barcode of Life Project (iBOL), has CO1 barcodes for 54% of elasmobranchs and 62% of holocephalans ( http://www.fishbol.org, accessed July 24, 2014). Recently, whole mitochondrial sequences are increasingly popular for their increased granularity when resolving branches of phylogenetic trees ^1^. Whole mitochondrial genome sequences currently are available for 72 species of sharks, skates, rays and chimaeras. These sequences are accessible in the GenBank, EMBL and DDBJ databases summarized in Table 5 ^58^.

**Table 5.** Whole mitochondrial sequences for chondrichthyan fishes.

Accessions						
BioProject	NCBI Ref_seq	GenBank	Organism	bp	^*^G+C	Date
Holocephali						
Chimaeriformes						
PRJNA50265	NC_014281.1	HM147135.1	*Callorhinchus callorynchus*	16758	34	21-Oct-10
PRJNA50271	NC_014284.1	HM147136.1	*Callorhinchus capensis*	16760	34.1	21-Oct-10
PRJNA50273	NC_014285.1	HM147137.1	*Callorhinchus milii*	16769	33.7	21-Oct-10
PRJNA11978	NC_003136.1	AJ310140.1	*Chimaera monstrosa*	18580	38.6	14-Nov-06
PRJNA50279	NC_014288.1	HM147138	*Chimaera fulva*	21336	38.2	19-Oct-10
PRJNA50287	NC_014292.1	HM147140.1	*Harriotta raleighana*	18024	42.5	19-Oct-10
PRJNA50283	NC_014290.1	HM147139.1	*Hydrolagus lemures*	21233	39.4	19-Oct-10
PRJNA50289	NC_014293.1	HM147141.1	*Rhinochimaera pacifica*	24889	41.6	19-Oct-10
Elasmobranchii: Batoids (rays and skates)						
Myliobatiformes						
PRJNA247653	NC_024102.1	KJ617038.1	*Gymnura poecilura*	17874	45.1	7-May-14
PRJNA239601	NC_023525.1	KF751650.1	*Himantura granulata*	17657	39.1	25-Feb-14
PRJNA229016	NC_022837.1	KF482070.1	*Aetobatus flagellum*	20201	40.9	3-Nov-13
PRJNA198706	NC_021132.1	KC526959.1	*Dasyatis akajei*	17658	40.4	10-Mar-14
PRJNA190131	NC_020352.2	KC196067.2 KC633222.1	*Dasyatis bennetti* *Dasyatis bennetti*	17668 17717	40.2 40.1	22-Jul-13 20-Feb-14
PRJNA182669	NC_019643.1	JX524174.1	*Dasyatis zugei*	18264	36.6	24-May-13
PRJNA15549	NC_007230.1	AY597334.1	*Plesiobatis daviesi*	17514	41.9	20-Mar-07
PRJNA232219	NC_023116	KF709642.1	*Potamotrygon motoro*	17448	43.3	14-Jan-14
PRJNA177278	NC_018784.1	JX392983.1	*Mobula japanica*	18880	37.4	18-Jan-13
PRJNA212605	NC_021767.1	KC992792.1	*Neotrygon kuhlii*	18039	39.5	17-Jul-13
PRJNA182647	NC_019641.1	JX827260.1	*Taeniura meyeni*	17638	41.6	8-Nov-13
Rajiformes						
PRJNA239623	NC_023505.2	KF318309.2	*Dipturus kwangtungensis*	16912	41.6	13-Mar-14
PRJNA81399	NC_016429.1	JQ034406.1	*Leucoraja erinacea*	16724	40.3	28-Nov-11
PRJNA13984	NC_007173.1	AY525783.1	*Okamejei kenojei*	16972	42.4	15-Jun-05
PRJNA11877	NC_000893.1	AF106038.1	*Amblyraja radiata*	16783	40.3	22-Apr-09
PRJNA214406	NC_021964	KC914434.1	*Raja rhina*	16910	41.4	11-Sep-13
PRJNA214407	NC_021963.1	KC914433.1	*Hongeo koreana*	16905	42.2	11-Sep-13
PRJNA244226	NC_023944.1	KF648508.1	*Zearaja chilensis*	16909	41.1	1-May-14
Rhinopristiformes						
PRJNA228994	NC_022821.1	KF381507.1	*Pristis clavata*	16804	39.8	13-Nov-13
PRJNA229000	NC_022841.1	KF534708.1	*Rhinobatos hynnicephalus*	16776	40.3	13-Nov-13
PRJNA244205	NC_023951.1	KJ140136.1	*Rhinobatos schlegelii*	16780	39.6	6-Apr-14
Elasmonbranchii: Selachii (sharks)						
Carcharhiniformes						
PRJNA246074	NC_024055.1	KF728380.1	*Carcharhinus acronotus*	16719	38.4	29-Apr-14
PRJNA244183	NC_023948.1	KF956523.1	*Carcharhinus amblyrhynchoides*	16705	38.2	6-Apr-14
PRJNA239607	NC_023522.1	KF646785.1	*Carcharhinus leucas*	16704	37.4	25-Feb-14
PRJNA252486	NC_024284.1	KJ720818.1	*Carcharhinus melanopterus*	16706	38.6	7-Jun-14
PRJNA193929	NC_020611.1	KC470543.1	*Carcharhinus obscurus*	16706	38.6	8-Nov-13
PRJNA239626	NC_023521.1	KF612341.1	*Carcharhinus sorrah*	16707	38.9	25-Feb-14
PRJNA217222	NC_022193.1	KF111728.1	*Galeocerdo cuvier*	16703	36.9	31-Oct-13
PRJNA236275	NC_023361.1	KF646786.1	*Glyphis garricki*	16702	39.2	13-Jan-14
PRJNA212606	NC_021768.2	KF006312.2	*Glyphis glyphis*	16701	39	25-Jul-14
PRJNA239588	NC_023527.1	KF889325.1	*Mustelus griseus*	16754	39	25-Feb-14
PRJNA11875	NC_000890.1	AB015962.1	*Mustelus manazo*	16707	38.3	8-Apr-00
PRJNA228986	NC_022819.1	KF356249.1	*Prionace glauca*	16705	37.5	13-Nov-13
PRJNA226181	NC_022735.1	AB560493.1	*Pseudotriakis microdon*	16700	36.4	29-Oct-13
PRJNA168394	NC_018052.1	JQ693102.1	*Scoliodon macrorhynchos*	16693	37	31-Mar-14
PRJNA11849	NC_001950.1	Y16067.1	*Scyliorhinus canicula*	16697	38	18-Apr-05
PRJNA226138	NC_022679.1	JX827259.1	*Sphyrna lewini*	16726	39.5	8-Nov-13
Orectolobiformes						
PRJNA163947	NC_017882.1	JQ434458.1	*Chiloscyllium griseum*	16755	36.1	6-Mar-12
PRJNA37667	NC_012570.1	JX162601.1	*Chiloscyllium plagiosum*	16725	37.4	25-Jul-12
PRJNA81281	NC_016686.1	JQ082337.1	*Chiloscyllium punctatum*	16703	36.8	31-Mar-14
PRJNA217221	NC_022148.1	KF111729.1	*Orectolobus japonicus*	16706	37.3	19-Sep-13
PRJNA238093	NC_023455.1	KF679782.1 KC633221	*Rhincodon typus* *Rhincodon typus*	16875 16928	37.1 37.1	19-Mar-14 31-Mar-14
Lamniformes						
PRJNA239610	NC_023520.1	KF569943.1	*Carcharias taurus*	16773	39.5	5-Feb-14
PRJNA221185	NC_022415.1	KC914387.1	*Carcharodon carcharias*	16744	40.8	31-Oct-13
PRJNA232870	NC_023266.1	KF597303.1	*Cetorhinus maximus*	16670	40.6	14-Jan-14
PRJNA226140	NC_022691.1	KF361861.1	*Isurus oxyrinchus*	16701	43.2	28-Sep-13
PRJNA247657	NC_024101.1	KJ616742.1	*Isurus paucus*	16704	43.8	7-May-14
PRJNA252473	NC_024269.1	KF962053.1	*Lamna ditropis*	16699	41.8	30-May-14
PRJNA207613	NC_021442.1	KC702506.1	*Megachasma pelagios*	16694	36.7	13-May-13
PRJNA33525	NC_011825.1	EU528659.1	*Mitsukurina owstoni*	17743	38.8	29-Dec-08
PRJNA228992	NC_022822.1	KF412639.1	*Alopias pelagicus*	16692	38.6	18-Dec-13
PRJNA207614	NC_021443.1	KC757415.1	*Alopias superciliosus*	16719	39.3	26-Jun-13
Heterodontiformes						
PRJNA11979	NC_003137.1	AJ310141.1	*Heterodontus francisci*	16708	39.9	14-Nov-06
PRJNA209901	NC_021615.1	KC845548.1	*Heterodontus zebra*	16720	40	18-Jun-13
Squaliformes						
PRJNA246067	NC_024059.1	KJ128289.1	*Cirrhigaleus australis*	16543	38.8	29-Apr-14
PRJNA226141	NC_022734.1	AB560492.1	*Somniosus pacificus*	16730	39.3	29-Oct-13
PRJNA11856	NC_002012.1	Y18134.1	*Squalus acanthias*	16738	38.8	18-Apr-05
Squatiniformes						
PRJNA252467	NC_024276.1	KJ619663.1	*Squatina japonica*	16689	37.9	4-Jun-14
Pristiophoriformes						
PRJNA247682	NC_024110.1	AB721306.1	*Pristiophorus japonicus*	18430	44.5	10-May-14
Hexanchiformes						
PRJNA226134	NC_022732.1	AB560490.1	*Hexanchus griseus*	17223	36.3	29-Oct-13
PRJNA226149	NC_022733.1	AB560491.1	*Hexanchus nakamurai*	18605	36.3	29-Oct-13
PRJNA226155	NC_022730.1	AB560488.1	*Heptranchias perlo*	18909	35.9	29-Oct-13
PRJNA226147	NC_022729.1	AB560487.1	*Chlamydoselachus anguineus*	17314	35	29-Oct-13
PRJNA226123	NC_022731.1	AB560489.1	*Notorynchus cepedianus*	16990	38.2	29-Oct-13

*Metazoan Mitochondrial Genomes Accessible dataset Metamiga ( http://amiga.cbmeg.unicamp.br/)

## Chondrichthyan Tree of Life

Currently, molecular data for cartilaginous fishes is being collected as part of the Chondrichthyan Tree of Life project ( http://sharksrays.org). The project website currently includes 5 elements: 1) an interactive phylogenetic tree ^55^; 2) scientific illustrations of specimens; 3) range information for all extant species; 4) interactive comparative anatomy through segmented CT scan data; and 5) DNA sequence for 1265 single copy orthologous genes ^59^. Project data will be available in public databases as well as through the project website once collection and analysis is complete (personal communication, Gavin Naylor, Medical University of South Carolina).

## Protein databases

Given the improved technologies to characterize full-length transcripts using RNA-Seq, there are increasingly more protein sequence data for chondrichthyans. The UniProt Consortium, consists of groups from the European Bioinformatics Institute (EBI), the Swiss Institute of Bioinformatics (SIB) and the Protein Information Resource (PIR). The consortium maintains the UniProt Knowledgebase (UniProtKB), a comprehensive and standardized catalogue of protein sequences and functional annotation knowledgebase ^60^. Proteins with UniProtKB accessions are first automatically annotated, unreviewed UniProtKB/TrEMBL entries that progress to UniProtKB/Swiss-Prot entries following curator review. Among Chondrichthyes, there are 12 UniProtKB/Swiss-Prot and 20,170 UniProtKB/TrEMBL entries for holocephalans and 264 UniProtKB/Swiss-Prot and 6,299 UniProtKB/TrEMBL entries for elasmobranchs in Release 2014_07 of 09-Jul-2014 of the knowledgebase ( Table 1). An unidentified fin sample accounts for 16 UniProtKB/TrEMBL entries that are not included in either Holocephali or Elasmobranchii. PDB, an archive of protein macromolecular structural data, has 178 entries for Chondrichthyes, all elasmobranchs ^61^. Of these, 76% are derived from 2 species from a single family, Torpediniformes, the electric rays, and in total only 10 species are represented in PDB.

**Figure 4.**
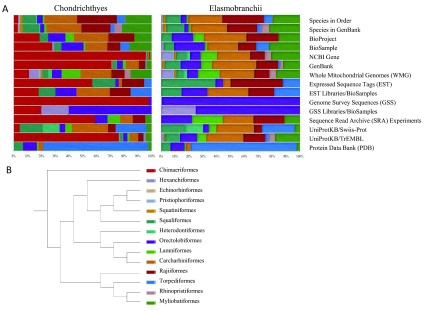
A survey of public data and phylogeny for chondrichthyan orders. **A**. The 14 orders of chondrichthyan fish and their relative distribution in public nucleotide and protein databases for Chondrichthyes and Elasmobranchii are shown individually. The species distribution for each Order and GenBank are similar indicating sequence data has been collected for a broad range of chondrichthyans. For Chondrichthyes, the elephant shark genome project data contributes the majority of the data in NCBI Gene, GenBank, Genome Survey Sequence (GSS), and the Short Reach Archive (SRA) databases. The NCBI GSS, GSS libraries, and Protein Data Bank (PDB) are the least diverse with representation of 1–6 of the 14 Orders. The color of each Order as represented in the bar chart is included in the cladogram key with left to right in the bar chart corresponding with top to bottom in the cladogram. **B**. A cladogram of Chondrichthyes illustrates the phylogeny relationship between the 14 Orders. The color code associated with each Order appears consecutively in the bar chart.

The distribution of data in NCBI databases, PDB, and UniProtKB for chondrichthyan orders is shown in Figure 4. When order Chimaeriformes is included ( Figure 4A) the distributions are disproportionate due to the large volume of annotated sequence data from the elephant shark genome. The distributions are repeated exclusively for elasmobranchs. To understand if the data distribution is representative of the number of species in each order, a species distribution is included in each chart. A cladogram ( Figure 4B) is linked to the chart legend and illustrates the phylogeny between chondrichthyan orders.

## SkateBase

SkateBase ( http://skatebase.org) is the public portal for the little skate genome project and is a valuable collection of data and learning resources. The NEBC little skate genome project team hosted three week-long workshops and a mitochondrial genome annotation jamboree with the goal of using the project data to develop a bioinformatics aware workforce and foster collaborative and distributed big data research. The lecture materials and worked annotation examples are included at SkateBase for educational use ( http://skatebase.org/workshops). The project vitae contains an overview and timeline of the genome project effort along with key personnel, project related publications and presentations, the curation team, and citation information for researchers utilizing the resource in their publication. A Gene Table currently represents manually curated genes derived from workshops and curriculum with extensive annotation evidence. The number of gene entries will continue to grow through usage and expansion of the SkateBase educational modules. Plans to update the annotation interface to enable community annotation by domain experts is planned for the future.

SkateBase provides links to web resources with chondrichthyan data including the Chondrichthyan Tree of Life, Elephant Shark Genome Project ( http://esharkgenome.imcb.a-star.edu.sg), the first described genome for a chimaera ^43^, and Vertebrate TimeCapsule, ( http://transcriptome.cdb.riken.go.jp/vtcap), a project that aims to develop a gene database to represent evolution and development for vertebrates and currently includes transcriptome data for a hagfish ( *Eptatretus burger*), shark ( *S. torazame*) and birchir ( *Polypterus senegalus*) ^62^. SkateBase data is linked locally as well as from NCBI in the Gene Expression Omnibus (GSE26235), GenBank (AESE010000000) and Sequence Read Archive (SRA026856) to ensure convenient and easy access. A link to the American Elasmobranch Society ( http://www.elasmo.org), a non-profit organization with the mission of advancing the scientific study of living and fossil sharks, skates, rays, and chimaeras and promoting education, conservation, and wise utilization of natural resources, connects domain scientists to the little skate genome project.

SkateBase data includes embryonic transcriptomes for three chondrichthyan species, a chimaera, *C. milii*, a shark, *S. canicula* and the little skate, *L. erinacea* as well as the first draft of the little skate genome. The assembled skate genome sequence gave a single high-coverage contiguous sequence that represented the entire length of the mitochondrial genome. The mitochondrial genome was subsequently annotated as part of a Jamboree in 2011 ^63^. The annotated sequence is represented by the NCBI Reference Sequence (RefSeq) project, accession NC_016429, and provides extensive information for each gene.

Whole embryos were used to build the transcriptome libraries available at SkateBase ^35^. Two *C. milii* embryos, stage 32, were combined and used to build a chimaera library. The transcriptome library for *S. canicula* was assembled from six pooled embryos, stages 24–30. The embryonic skate transcriptome library was assembled using six pooled embryos ranging in stage from 20–29. This combination of stages encompasses a large portion of the developmental period for these fishes and represents a catalog of genes important for organogenesis of all or part of every physiological system. Early developmental events are similar for nearly all elasmobranchs regardless of reproductive mode or adult body form enabling the data to be useful for more than just the specific species from where it was derived ^64^. Since all three embryonic transcriptomes contain a similar stage embryo direct comparison for temporal expression patterns is possible. Skatebase includes tools for data investigation, SkateBLAST, a sequence retrieval tool, Skate Contig Lookup, and genome browsers for three skate whole mitochondrial sequences, *L. erinacea*, the thorny skate, *Amblyraja radiata,* and, the ocellate spot skate, *Okamejei kenojei*. Skatebase contains resources that can be used for teaching and research purposes. As an example, two use cases follow, one for sequence or homology based research and the other for education.

## SkateBLAST

A common task for researchers is searching for genes of interest in a genome or transcriptome. Knowledge of the gene sequence at the DNA or RNA level is needed for many different studies, including phylogenetic analysis or designing primers for quantitative PCR gene expression studies. Here we describe the major steps necessary to identify relevant sequences for a gene of interest using the BLAST sequence similarity tool at SkateBase. SkateBase features a web interface to BLAST, named SkateBLAST, that builds upon the ViroBLAST package version 2.2 ^65^, with custom modifications allowing parallel cluster-based execution of queries and enhanced display of results. The overall workflow consists of a) entering a query sequence and selecting the database to search; b) evaluating the alignments returned; c) retrieving the sequence from one of the SkateBLAST databases; and d) checking to make sure that the retrieved sequence aligns best to the query sequence. The following description provides a brief tutorial on the overall workflow while describing tools at SkateBase.

**Figure 5.**
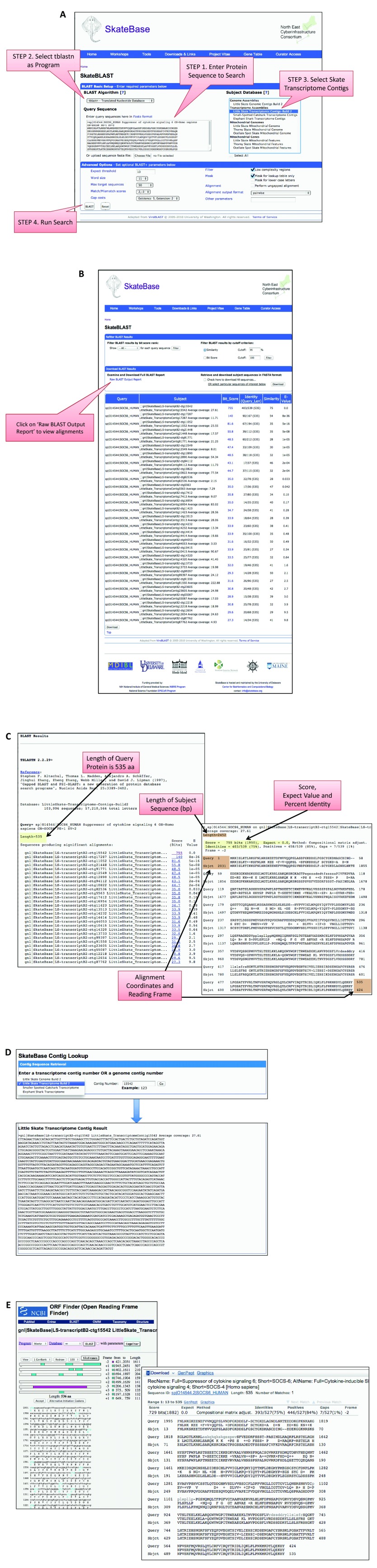
Example of using SkateBase and NCBI resources to find transcriptome data for SOCS6. **A**. SkateBLAST query form showing the four steps to align the UniProt sequence for human SOCS6 (O14544) against the skate embryonic transcriptome using tblastn. Step 1 is to enter the sequence in FASTA format. The second step is to choose the tblastn program that will align the query protein sequence against translated sequences in all six possible reading frames. The third step is to select the embryonic transcriptome as the sequence database to search. The fourth step is to launch the search. **B**. The complete BLAST output can be accessed by clicking the “Inspect BLAST output” link at the top of the summary report page. This is necessary to examine the sequence alignments. **C**. Four important fields in the output should be examined carefully to interpret the alignments and determine which returned alignment best represents the skate ortholog to SOCS6. First, the alignment score, E-value, alignment length and percent identity can be used to interpret the overall alignment significance. Alignment coverage with respect to the query protein sequence and the subject transcriptome sequence can be interpreted by comparing the alignment coordinates to the length of the query protein sequence and length of the transcriptome sequence. In this example, the entire query protein sequence is covered by this transcriptome sequence. **D**. The SkateBase Contig Lookup tool can be used to retrieve the transcriptome sequence found in the SOCS6 tblastn search in FASTA format. Sequences from the skate genome assembly or the skate, *S. canicula* or *C. milii* transcriptome assemblies can be retrieved using this tool. **E**. Output from the NCBI ORF Finder tool showing a 536aa ORF in the skate transcriptome contig that best represents SOCS6 (left). Alignment from blastx search of the skate transcriptome sequence (contig 15542) against human UniProt using NCBI BLAST to validate that the contig aligned best to human SOSC6 rather than another human gene.

Figure 5 demonstrates the use of SkateBLAST to find expressed sequences for the gene, suppressor of cytokine signaling 6 ( *SOCS6*). *SOCS6* is a E3 ubiquitin ligase that interacts with c-KIT to suppress cellular proliferation through its SH2 domain ^66^. The first step to identify *SOCS6* in the skate transcriptome begins with entering the protein sequence for human SOCS6 that was obtained from UniProt and searching this sequence against the skate transcriptome using the tblastn program. The next step is to evaluate the alignments to determine which transcriptome sequences best represent *SOCS6*. When interpreting the pairwise alignments from SkateBlast as in any BLAST tool, it is important to examine: a) alignment statistics; b) alignment coverage; and c) presence of protein domains that you may expect to be conserved. The alignment statistics are reported to ascertain whether you would expect the given alignment by chance or not. There are three key alignment statistics, the expectation (E)-value, percent identity and alignment length. The E-value represents the probability that you would expect an alignment with that alignment score or better by random chance, thus the lower the E-value, the better the alignment. Conversely, the greater the percent identity (percent identical sequence) and alignment length, the more similar the two sequences are assumed to be. Alignment coverage with respect to the query or subject sequence (alignment length divided by the length of the query or subject sequence) can also be an important consideration, as low coverage suggests that important regions of one or both sequences may not be represented in the alignment. Finally, there may be particular sequence features, such as protein domains, that you would expect to find in the alignment. If those domains are missing, then it suggests that you have a partial or misleading alignment.

Once a transcriptome sequence of interest, such as contig15542, is identified in the SkateBLAST results, you must do a reciprocal search of that sequence against a database of protein sequences to confirm that the sequence aligns best to your gene of interest. You can retrieve the full sequence directly from the BLAST tool or using the Skate Contig Lookup tool ( Figure 5D): a) specify the transcriptome that you had originally searched using SkateBLAST; b) enter the sequence identification or contig number is entered into the query box; and c) select the ‘GO’ button. The user can copy the returned sequence and use it for further exploration of sequence homology at NCBI or similar databases.

## SkateBase classroom use case: teach concepts of gene and protein annotation

SkateBase includes valuable teaching resources derived from the project workshops on gene and protein annotation. Infrastructure for sequence annotation was developed and modules for use in teaching are available. Access to the teaching modules is through the Curator Access link from the homepage and permission is granted by request using the email link at the bottom of each page, info@SkateBase.org. Once successfully logged into the site, access to pre-computed blast results, guides and examples, annotation forms, and links to external tools helpful for sequence analysis are available. Gene annotation begins with a transcriptome contig identified through a SkateBlast search as illustrated above. The portion of the transcript that codes for protein is identified using an open reading frame or ORF finder tool. Annotation follows a workflow where complimentary sequences from the transcriptome and genome are aligned allowing annotation of both sequences using Sequence Ontology vocabulary ^67^. The evidence is recorded in an annotation form that records information about the annotator and sequences and includes a comment box for questions and comments between students and teachers or curators and annotators. The annotation form records the pairwise alignment of the transcriptome and genome contigs, notes concerning mismatches or gaps, as well as output from the ORF tool. The untranslated regions (UTR) at the beginning and end of each sequence, 5’UTR and 3’ UTR regions, as well as the intron/exon structure for the genomic contig and CDS for the transcriptomic contig are recorded in the Gene Annotation Form. When completing the Gene Annotation Form, the appropriate activity must be selected and can be customized to specify the user’s course ID, institution or workshop title to track annotation history. Protein annotation uses the rapid annotation interface for proteins, RACE-P, developed by the PIR. A UniProt accession number is required to initiate a new annotation form. The form is composed of 6 blocks of information, protein information, gene information, a bibliography, Gene Ontology (GO), computational analysis using tools such as Pfam ^68^, TMHMM ^69^, SignalP ^70^, COILS ^71^, NetPhos ^72^ and EMBOSS ^73^, and protein family evidence.

## Discussion

**Figure 6.**
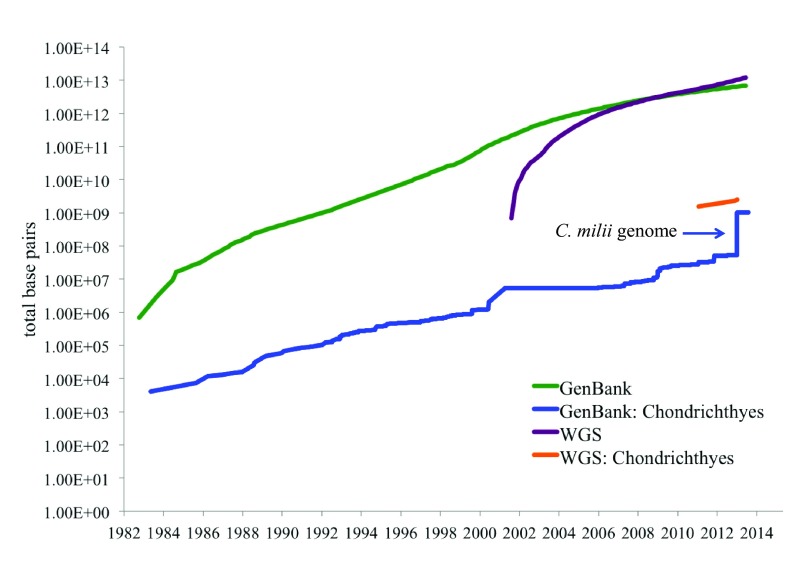
GenBank and WGS data trends for Chondrichthyes and all taxa. GenBank is the National Institutes of Health (NIH) genetic sequence database and together with the DNA Databank of Japan (DDBJ) and the European Molecular Biological Laboratory (EMBL) comprise the International Nucleotide Sequence Database Collaboration (INSDC). The cumulative base pair total for all taxa as well as chondrichthyan only data are given versus time for GenBank and Whole Genome Shotgun (WGS) data. The Elephant Shark Genome Project is responsible for the spike in chondrichthyan GenBank in 2011. The little skate and elephant shark genome projects are currently the only two WGS datasets (yellow line).

The volume of data in GenBank continues to grow exponentially, doubling nearly every 18 months. The first sequences for chondrichthyes appeared in 1983 and the overall data trend for chondrichthyans is similar to all of GenBank with three exceptions. First, the rate of increase is less than GenBank. Second, the number of sequences deposited during the first decade of the 21 ^st^ century was nearly stagnant in comparison. Third, a large spike is observed in late 2012 attributed to the Elephant Shark Genome Project data ( Figure 6). Molecular data is increasingly important for all aspects of research utilizing chondrichthyan fishes ^74^. It can be a forensic tool to understand species when fins are landed without carcass and ensure protected species and quotas are respected ^75– 79^. For migrating species molecular data serves as a surrogate to classical tagging data to understand population structure and range ^80– 83^. In studies of evolution, molecular data provides estimates of divergence time and supplements morphological and ecological traits as the basis for a phylogeny. The benefits and uses of molecular data for these fishes are limited only by the amount of data available. SkateBase provides the only genomic data publically available for an elasmobranch in addition to embryonic transcriptomes, data tools, and educational resources.

Sequencing projects require significant funding and personnel commitments but generate a large amount of information that can be translated to knowledge by domain experts. The efficiency of this process is affected most by allowing the scientific community to access the data. The value of data sharing can be measured by the number of publications that result from its distribution. To date, 19 publications in peer-reviewed journals have used data derived from SkateBase ( http://skatebase.org/vitae). Molecular data are the means to investigate genes and develop reagents for gene expression studies by PCR or *in situ* hybridization. Small scale sequencing efforts that generate limited or fragmented data often get deposited to hard disks and remain ‘buried’ and out of reach. Efforts to deposit this data at public sequence repositories are encouraged to build the foundation of data required to describe this dynamic and ancient clade of fishes. We invite investigators to contact the authors in an effort to survey the volume of private data available for potential distribution through SkateBase.

The transcriptome data at SkateBase serves as a platform to teach molecular techniques, technologies, and bioinformatics in the context of studying elasmobranchs. As next generation sequencing (NGS) technologies evolve it is important for scientists and students to understand how the sequence was generated and caveats of workflow for each data type in order to recognize errors and customize analysis algorithms. The educational materials and infrastructure at SkateBase have been used by University of Delaware, Georgetown University, MDI Biological Laboratory, University of Maine at Machias, University of Rhode Island, and most recently the Virginia Institute of Marine Science to teach gene and protein annotation concepts. We invite and look forward to continued expansion of the SkateBase educational platform as we refine the infrastructure and expand the data available for investigation through continued sequencing efforts.
