# Supplementary material for: SkateBase, an elasmobranch genome project and collection of molecular resources for chondrichthyan fishes
Source: F1000Res. 2014 Aug 12;3:191. [Version 1] doi: 10.12688/f1000research.4996.1 (PMC4184313; doi:10.12688/f1000research.4996.1)
Supplement: Peer review history

# Peer review: Referee response for version 1

**DOI:** 10.5256/f1000research.5333.r5798

**Published:** 2014-09-01

**Authors:** Ed Heist

**Affiliations:** 1 Department of Zoology, Southern Illinois University, Carbondale, USA

This manuscript has two components: a description of current genomic, proteomic, and transcriptomic resources for chondrichthyan fishes and a description of the SkateBase project.  Of the two, I would rather have seen more emphasis on SkateBase since the rate at which new data are being generated will make the survey data presented in the manuscript increasingly incomplete.  Nevertheless I think it is a good paper worthy of publication and SkateBase seems to be a valuable resource.

Specific comments include:

Abstract – capitalize Sarcopterygii, Actinopterygii misspelled.  Throughout the manuscript there are numerous inconsistencies in capitalization, e.g. “order Chimaeriformes” and “Fish barcode of life (FISH-BOL) a working group of the International Barcode of Life Project (iBOL)” both on page 8.

Introduction – In the first sentence replace “Chondrichthyan fishes” with “Living chondrichthyan fishes.”

Paragraph 2 – The text implies that the only benefits of chondrichthyans are ecotourism and fisheries.  Perhaps a mention of ecological services (e.g. food-web dynamics) or other benefits of chondrichthyan conservation would be appropriate here.

Paragraph 4 – “asexual” parthenogenesis seems redundant.

Paragraph 5  – perhaps some more details on the distribution, life history, reproductive mode, genome size etc. of little skate is in order.  Why is the geographic location of the species relevant?

Figure 1 is hard for me to decipher.  I can’t figure out what the two separate outer rings indicate and why the spacing between them is different for the orders vs. the families, genera, and species.  I really think this figure is too complex and tries to cram too much information that is peripheral to the goals of the paper into a single figure.  I may be old fashioned, but I think a table that listed the numbers of families, genera, and species would have been sufficient.  There also needs to be a citation on the source of the information as the taxonomy and number of recognized species of chondrichthyans is changing. 

Figure 4B – A citation is needed for the cladogram.

I have read this submission. I believe that I have an appropriate level of expertise to confirm that it is of an acceptable scientific standard.

# Peer review: Referee response for version 1

**DOI:** 10.5256/f1000research.5333.r5793

**Published:** 2014-08-26

**Authors:** Kevin Feldheim

**Affiliations:** 1 Pritzker Laboratory for Molecular Systematics and Evolution, Field Museum of Natural History, Chicago, USA

The authors describe a publicly accessible database SkateBase, a public site for the little skate genome project. This paper is well written, and I have just a few comments and suggestions:

 

- Why was the little skate picked for genome size? Is it small and manageable? Is it a typical size compared to other chondrichthyans?
- Page 3, 1 ^st^ column: change “compliance with fishing quotas” to “maintain compliance with fishing quotas”.
- Page 3, 2 ^nd^ column: Heterodontiformes is misspelled (Heterdontiformes).
- Page 3, 2 ^nd^ column: Lamniformes is misspelled (Lamniormes).
- Page 3, under “Existing resources” I suggest including Chimaeriformes here as well.
- Page 7: *Heterodontus* is misspelled ( *Heterdontus*).
- Page 8: species name for hagfish should be *burgeri* (not *burger*).
- Page 8: The last three sentences under the “Protein databases” section can be incorporated into the legend for Figure 4.
- Page 8: bichir is misspelled (birchir).
- Page 8: change “SkateBase data is linked” to “SkateBase data are linked” and “SkateBase data includes” to “SkateBase data include”.
- Page 19, 1 ^st^ column: change “this data” to “these data”.

 

Figures and Tables:

 

- In the figure 1 legend — most of this information is stated in the text. I suggest keeping the sentence: “The distribution of chondrichthyan species in each of the 14 orders is shown relative to the total number of species, genera and families for the clade.” I also suggest explaining the figure in a little more detail. What do the numbers mean? Do the colors represent anything?
- Figure 2 legend: “is” should not be italicized.
- Table 1: What do the numbers in the columns mean? Are these numbers of entries?
- Table 2: Contigs for *L. erinacea *is 2,62,265. Also, under N50, change 1466 to 1,466.
- Figure 4: It is difficult to tell apart colors for the following pairs:
  ​
  - Squatiniformes and Carcharhiniformes
  - Lamniformes and Myliobatiformes
  - Chimaeriformes and Rajiiformes
- This can be rectified by using black, white and yellow for three of the above orders in the figure.
- Figure 5 legend: I suggest changing “Four important fields in the output should be examined carefully to interpret the alignments and determine which returned alignment best represents the skate ortholog to SOCS6. First, the alignment score, E-value, alignment length and percent identity can be used to interpret the overall alignment significance.” to “Four important fields in the output should be examined carefully to interpret the alignments: the alignment score, E-value, alignment length and percent identity.”

I have read this submission. I believe that I have an appropriate level of expertise to confirm that it is of an acceptable scientific standard.
